# Sensitivity Analysis for Not-at-Random Missing Data in Trial-Based Cost-Effectiveness Analysis: A Tutorial

**DOI:** 10.1007/s40273-018-0650-5

**Published:** 2018-04-20

**Authors:** Baptiste Leurent, Manuel Gomes, Rita Faria, Stephen Morris, Richard Grieve, James R. Carpenter

**Affiliations:** 1 Department of Medical Statistics, London School of Hygiene and Tropical Medicine, Keppel Street, London, WC1E 7HT UK; 2 Department of Applied Health Research, University College London, London, UK; 3 Centre for Health Economics, University of York, York, UK; 4 Department of Health Services Research and Policy, London School of Hygiene and Tropical Medicine, London, UK; 5 MRC Clinical Trials Unit at University College London, London, UK

## Abstract

Cost-effectiveness analyses (CEA) of randomised controlled trials are a key source of information for health care decision makers. Missing data are, however, a common issue that can seriously undermine their validity. A major concern is that the chance of data being missing may be directly linked to the unobserved value itself [missing not at random (MNAR)]. For example, patients with poorer health may be less likely to complete quality-of-life questionnaires. However, the extent to which this occurs cannot be ascertained from the data at hand. Guidelines recommend conducting sensitivity analyses to assess the robustness of conclusions to plausible MNAR assumptions, but this is rarely done in practice, possibly because of a lack of practical guidance. This tutorial aims to address this by presenting an accessible framework and practical guidance for conducting sensitivity analysis for MNAR data in trial-based CEA. We review some of the methods for conducting sensitivity analysis, but focus on one particularly accessible approach, where the data are multiply-imputed and then modified to reflect plausible MNAR scenarios. We illustrate the implementation of this approach on a weight-loss trial, providing the software code. We then explore further issues around its use in practice.

**Electronic supplementary material:**

The online version of this article (10.1007/s40273-018-0650-5) contains supplementary material, which is available to authorized users.

## Key Points for Decision Makers

**Table:** 

Cost-effectiveness analysis of randomised trials with missing data should assess the robustness of their findings to possible departures from the missing at random assumption.
Multiple imputation provides a flexible and accessible framework to conduct these sensitivity analyses.
Sensitivity analysis results should be reported in a transparent way, allowing decision-makers to assess the plausibility of their respective assumptions.

## Introduction

Cost-effectiveness analyses (CEA) of randomised trials are an important source of information to help decide which health care programmes to provide. A common issue is that there may be missing data, for example, because patients withdrew from the trials or failed to respond to study questionnaires, and this could result in biased findings and, ultimately, wrong decisions being taken.

There is now greater awareness that simple approaches, such as discarding the participants with missing data, are generally unsatisfactory [1–5]. The benefits of methods that make use of all the available data and offer valid inference under ‘missing at random’ (MAR) assumptions are now well recognised, and recent years have seen an increase in the use of such methods in CEA, in particular multiple imputation (MI) [6, 7].

A key concern, however, is that conditional on the observed data, the probability of cost-effectiveness data being missing may still depend on the underlying unobserved values, i.e. data may be ‘missing not at random’ (MNAR). For example, after adjusting for observed prognostic factors, the chances of completing quality-of-life questionnaires may depend on the patient’s (unobserved) quality-of-life status. This raises particular challenges to cost-effectiveness inferences because the analyst cannot formally choose between MAR and MNAR given the data at hand. Therefore, conducting sensitivity analyses to assess whether conclusions are robust to plausible departures from MAR is widely recommended [1, 2, 8–10], and these are particularly relevant for CEA which usually rely on patient-reported outcomes. However, a recent review has found that, in practice, cost-effectiveness studies rarely conduct such a sensitivity analysis [7]. We discussed this issue with stakeholders (academics from the University of York and the London School of Hygiene and Tropical Medicine analysing or reviewing cost-effectiveness evidence for health care decision making), and an important barrier that was identified was the lack of software tools and guidance to conduct these analyses.

This tutorial paper aims to address this gap by presenting an accessible framework and practical guidance to conduct sensitivity analysis for trial-based CEA with missing data. This builds on previous guidance on missing data in CEA [1, 3, 4], by focusing on sensitivity analysis approaches to address MNAR. This paper introduces different approaches to MNAR analyses, but focuses particularly on the implementation of pattern-mixture models using MI [11] as it was highlighted as the most accessible and flexible approach during our discussions with stakeholders. This tutorial assumes familiarity with the conduct of MI (under the MAR assumption), which has been covered elsewhere [3, 4, 12, 13].

The remaining sections of this paper are organised as follows. Section 2 provides a brief overview of the different approaches for MNAR analysis. Section 3 illustrates a framework for MNAR sensitivity analysis, based on a weight-loss trial, the Ten Top Tips (10TT) study. Section 4 discusses possible extensions to the proposed approach and further considerations for implementing it in practice.

## Overview of Missing Not at Random (MNAR) Analysis Methods

### Missing Data Mechanisms

The classification of the missing data mechanisms proposed by Little and Rubin [14] provides a useful context. Data are said to be missing ‘completely at random’ (MCAR), when missingness occurs for reasons unrelated to the analysis question, and hence independent of the variables of interest. In this case, the observed data are representative of the overall data and analysing the participants with complete data will give valid results. A less restrictive assumption is that the data are ‘missing at random’ (MAR), so that the probability of a value being missing may be dependent on observed data (e.g. intervention group, or participants’ age), but—given the observed data—independent of the underlying value itself. Finally, if, after taking into account the observed variables, the chance of observing the data is still associated with its value (for example, if, after controlling for preceding data, a patient is less likely to complete a health questionnaire when in poorer health), the data are said to be ‘missing not at random’ (MNAR, also called ‘informative’, or ‘non-ignorable’ missingness).

When missing data are MAR, valid conclusions can be drawn from the data available using an appropriate approach, such as MI [15]. MI has been widely recommended as a flexible, practical approach to handle missing data in CEA studies [1, 3–5, 12], and its uptake has been steadily increasing [6, 7]. The idea of MI follows from regression imputation (using the observed data to predict the missing values), but appropriately takes into account the uncertainty in the imputed values. To achieve this, missing observations are replaced by plausible values drawn from an appropriate predictive distribution of the missing values given the observed data. To reflect the fact that imputed values are estimated rather than known, and hence uncertain, this process is repeated several times to create several complete datasets. The analysis model is then fitted to each ‘complete’ dataset, and the results are combined for inference using Rubin’s MI rules [15], which recognise the uncertainty both within imputations (sampling uncertainty) and between imputations (uncertainty due to missing data).

Analysis under MNAR is more challenging, as it implies some relevant information is unobserved, and it requires additional untestable assumptions to proceed with the analysis. This naturally makes the MAR assumption the typical starting point for the primary analysis of clinical trials [16, 17]. However, because we cannot determine the true missing data mechanism, sensitivity analyses should be conducted in order to assess whether conclusions are robust to plausible departures from the MAR assumption [1, 2, 8–10].

### MNAR Modelling Frameworks

Various approaches have been proposed in the statistical literature to conduct analysis under MNAR. These vary according to how they formulate the MNAR model, how they fit this model, and how the unobserved parameters are informed and results reported as part of a sensitivity analysis strategy. Here, we briefly review some of the main MNAR modelling frameworks; for a more comprehensive description, see Molenberghs et al. [11]. There are two main ways to model possible departure from MAR: selection models and pattern-mixture models.

*Selection models* specify the mechanism by which the data are observed (or ‘selected’) as a function of the underlying data values [15, 18]. For example, ‘for each decrease of 0.1 in quality of life, the chance of being missing doubles’ formulates the MNAR problem in selection model terms. Selection models were commonly used in early work on informative missing data; an example in econometrics is Heckman’s selection model [19], which is used to address selection bias. They have the attractive feature that the missing data model can be directly incorporated into the analysis model, for example, using an inverse probability weighting approach [18, 20] or numerical integration [21]. However, selection models make untestable assumptions about the conditional distribution of the unobserved data, and results can be very sensitive to departure from these assumptions, as has been shown elsewhere [14, 22–24]. This limitation is particularly relevant for CEA studies, as the cost and effectiveness endpoints tend to be difficult to parametrise. Another disadvantage is that selection models formulate sensitivity analysis in a way that is not readily interpretable. For example, a typical sensitivity parameter is the (log-)odds ratio of how a unit change in the partially observed outcome affects the chances of observing the data. This specification makes the elicitation of such parameters challenging, as well as the interpretation and communication of the sensitivity analysis results.

*Pattern-mixture models*, on the other hand, formulate the MNAR problem in terms of the different distributions between the missing and observed data. The overall distribution of a variable is seen as a mixture of the distribution of the observed and the distribution of the missing values (‘pattern-mixture’) [18, 25]. For example, ‘participants with missing data have a 0.1 lower quality of life than those observed’ corresponds to a pattern-mixture formulation. Pattern-mixture models have received increasing attention over time [26], a key advantage being that they rely on more easily interpretable parameters [3, 18, 27–29]—such as the mean difference between missing and observed data—and have therefore been favoured in the context of clinical trial sensitivity analysis [30, 31]. Different approaches can be used to formulate and analyse pattern-mixture models, as we will see in the next section.

Other forms of MNAR modelling have also been proposed, but these can be seen as special cases of selection or pattern-mixture models. In shared-parameter models, the outcome and the missingness are linked through a latent (unobserved) variable [32]. They have been particularly used in the context of structural equation modelling. Another approach which is gaining interest for use in longitudinal trials is ‘reference-based’ or ‘controlled’ imputation, where missing data are assumed to follow a distribution borrowed from another trial arm [33]. This approach is yet to be explored in the CEA setting.

While any of the methods above would allow an appropriate assessment of departures from MAR, we will focus on the pattern-mixture approach in the remainder of this paper because (1) it allows for more interpretable parameters, hence making this approach more accessible and transparent; (2) it seems to be the main approach currently used in clinical trial sensitivity analysis [7, 34]; (3) our discussion with stakeholders confirmed this approach was also appealing in the CEA context; and (4) pattern-mixture models can be easily implemented using standard missing data methods, such as MI, and build naturally on the MAR analysis, as we will see below.

### Sensitivity Analysis with Pattern-Mixture Models

**Fig. 1:**
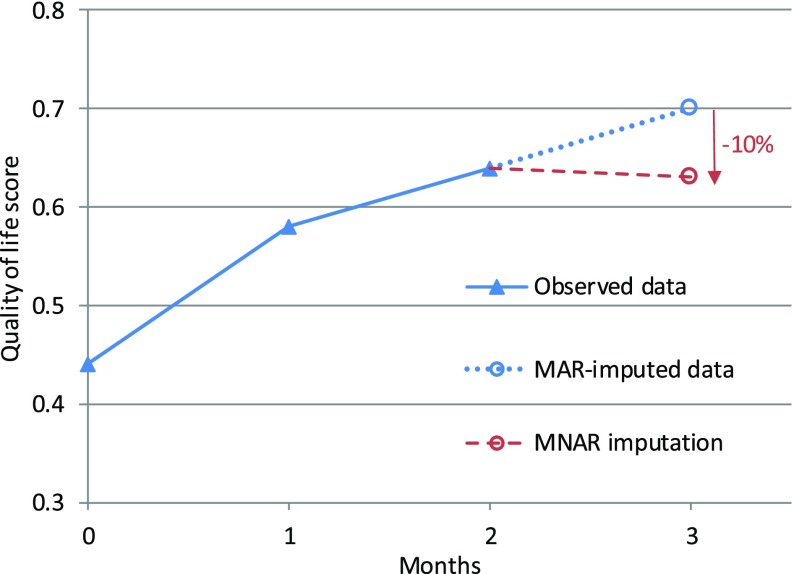
Example of pattern-mixture assumptions with rescaling. Quality-of-life score over time for a trial participant, where missing data are assumed to be 10% lower (*c *= 0.9) than would have been imputed under a missing at random assumption. *MAR* missing at random, *MNAR* missing not at random

An approach for MNAR sensitivity analysis that has often been suggested—under various forms—is to perform a pattern-mixture model with a parameter capturing how the distribution of the missing values $Y_{\text{miss}}$ could differ from the conditional distribution based on the observed data $Y_{\text{obs}}$ [15, 18, 30, 35]. This can be done, for example, by using an ‘offset’ parameter δ (delta) representing the average difference between the missing and observed values ($Y_{\text{miss}} = Y_{\text{obs}} + \delta ).$ An alternative modification is to use a multiplicative ‘scale’ parameter $c$, so that $Y_{\text{miss}} = Y_{\text{obs}} \times c$. For example, missing values could be assumed to be 10% lower than those observed, or $c = 0.9$. Figure 1 illustrates an example of such modelling with a rescaling parameter. In that example, a participant who drops out from the trial is assumed to have on average a 10% lower quality of life compared to a participant with similar characteristics who remained in the trial. Note that this parameter is not derived from the data, but is used to express one possible assumption about the (unknown) missing data mechanism.

Sensitivity analyses are then typically conducted over a range of plausible values for this parameter, assessing how different assumptions could result in different findings. Several approaches can be used to inform the values of the parameter in practice, and these are discussed further in Sect. 4.3. We also discuss in Sect. 4.2 alternative parametrisations that can be used to capture how missing and observed data might differ.

Several approaches have been proposed to fit pattern-mixture models, for example, within a Bayesian framework [28, 36] or as an arithmetic function of the observed estimates and using bootstrap or sandwich estimators to derive the standard errors [18, 28]. But a particularly convenient and flexible framework to fit these models is MI [11, 15, 26, 37]. An approach commonly adopted in practice consists of simply modifying multiply-imputed data to reflect possible departures from the MAR assumption [3, 7, 16, 38]. It involves the following steps:

1. Use MI to impute the missing values under an MAR assumption.
2. Modify the MAR-imputed data to reflect a range of plausible MNAR scenarios, for example, by multiplying the imputed values by $c$, or by adding $\delta .$
3. Analyse the resulting dataset as one would a usual multiply-imputed dataset, fitting the analysis model to each imputed dataset and combining the results using Rubin’s rules.

This approach is straightforward to implement in any statistical software, and allows the effect of different MNAR mechanisms on the conclusion to be easily assessed, as we will illustrate in the next section.

## Illustrative Application

### The Ten Top Tips (10TT) Trial

#### Overview of the Trial and Cost-Effectiveness Analysis

The 10TT trial was a two-arm, individually randomised, controlled trial of a weight-loss intervention for obese adults attending general practices in the UK [39]. The intervention comprised self-help material delivered by a practice nurse, providing the patients with a set of ten simple weight-control behaviours, with strategies to make them habitual. The participants randomised to the control arm received care as usual from their general practices.

The primary trial outcome was weight loss at 3 months, but participants were followed for 2 years to assess longer-term outcomes and cost-effectiveness. Health-related quality of life (HRQoL) was measured by EQ-5D-3L questionnaires [40, 41] completed during study visits at baseline and 3, 6, 12, 18 and 24 months, and quality-adjusted life years (QALYs) were derived by the ‘area under the curve’, combining both time and utilities [10]. Total costs were measured from the National Health Service (NHS) perspective over the 2-year study period and based on the intervention costs and the health resource use data collected from the practice records at the end of the trial. More details on the trial and CEA can be found in the respective publications [39, 42, 43].

#### Missing Data

**Fig. 2:**
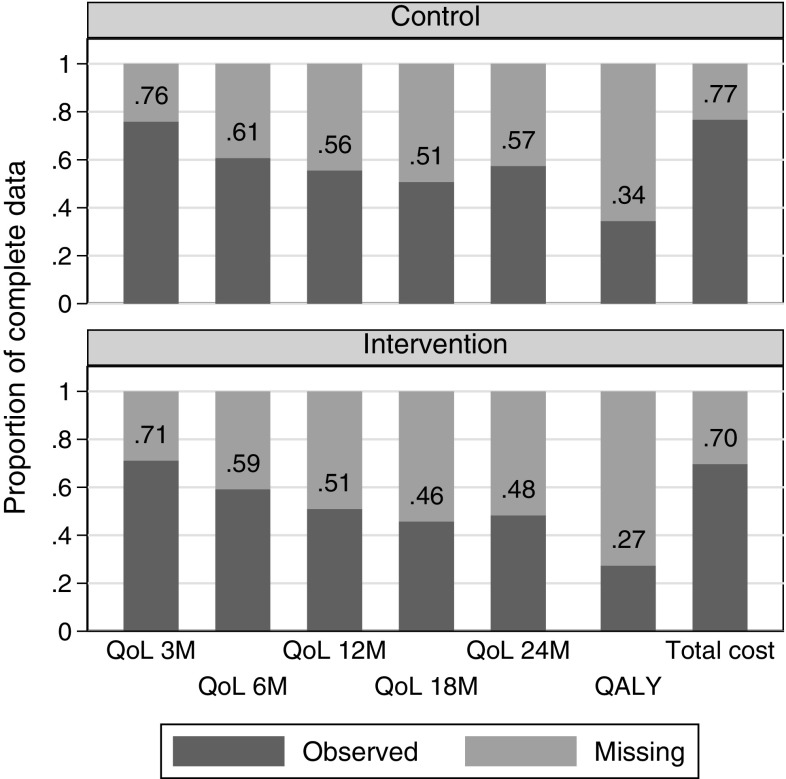
Proportion of complete cost-effectiveness outcomes in 10TT trial, by arm: QoL at each time point (3, 6, 12, 18, and 24 months), overall QALY, and total cost. *N* = 270 in control arm and 267 in intervention arm. *QALY* quality-adjusted life year, *QoL* quality of life, *10TT* Ten Top Tips

The trial recruited 537 participants, but only 313 (58%) completed the last follow-up at 2 years. Missing data were a major challenge for the CEA because only 31% of randomised participants had complete HRQoL and cost data. Missing data were mostly driven by missing EQ-5D data, from participants who had either withdrawn from the trial (76% of the missing HRQoL) or missed a follow-up appointment (24%). Resource use data were derived from the general practitioner records and were complete for 73% of the participants (all the health care data were missing for the remaining 27%). Details of the missing data by arm are shown in Fig. 2. Although non-significant, missing data appeared to be more common in the intervention arm (27 vs 34% of complete cases, *p* value = 0.075).

The primary CEA of the trial [43] was conducted under the MAR assumption, using MI to impute the missing cost and HRQoL values. It is, however, recognised in weight-loss trials that participants who drop out could be those with poorer outcomes [44]. This means that the chance of observing endpoints such as weight loss or HRQoL could be dependent on their actual value, i.e. data are likely to be MNAR. It is therefore important to assess the cost-effectiveness results under different assumptions regarding the missing data, including plausible MNAR mechanisms, as we will illustrate in Sect. 3.2.

#### Cost-Effectiveness Analysis Methods

The CEA conducted in this tutorial follows the main characteristics of the methods used for the trial’s primary CEA [43], with some simplifications made to allow a clear focus on the sensitivity analysis. Details of the analysis variables are presented in Online Appendix 1 [see the electronic supplementary material (ESM)]. Effectiveness was measured in QALYs, and costs were captured by the total health care use over the trial period (Sect. 3.1.1), as derived for the primary analysis [43]. A discount rate of 3.5% per year was applied to both cost and effect.

Results are presented in terms of incremental cost, incremental QALYs and incremental net monetary benefit (INMB) at a cost-effectiveness threshold of £20,000 per QALY. These were estimated alongside their 95% confidence intervals (CIs) using non-adjusted linear regression, comparing the 10TT arm to the control arm. Non-parametric bootstrap [45] was also used to produce the cost-effectiveness plane [46], representing the uncertainty in incremental cost and effect estimates, and the cost-effectiveness acceptability curve (CEAC) [47], representing the probability of 10TT being cost-effective at different thresholds. We focus on INMB rather than the incremental cost-effectiveness ratio (ICER) as the intervention was cost-saving. All the analyses were conducted in Stata version 15 [48].

### Sensitivity Analysis Example

In this section, we use the 10TT trial to illustrate MNAR sensitivity analyses using a pattern-mixture approach following MI, as described at the end of Sect. 2.3.

#### MNAR Scenarios Explored

Several approaches can be used to decide on the relevant MNAR scenarios for the sensitivity analyses, and this is discussed further in Sect. 4.3. In this example, we considered that the missing HRQoL data may be MNAR, while the MAR assumption is likely to hold for the missing cost data (MNAR costs are discussed in Sect. 4.1). It was postulated that patients who failed to complete an EQ-5D questionnaire at a specific follow-up assessment were likely to have been in relatively poorer health (Sect. 3.1.2). More specifically, we assumed patients’ HRQoL could be up to 10% lower (*c* = 0.9), compared to the MAR setting (*c *= 1). This sensitivity parameter *c* was allowed to differ by arm, with up to a 5% difference between the two arms (this reflects that the missing data mechanism may not be the same in the two arms, but that it is unlikely to be perfect MAR in one arm and strong MNAR in the other). This resulted in seven different MNAR scenarios, with *c *= 1.0, 0.95, or 0.9 for either arm (Table 1).

**Table 1:** Cost-effectiveness of 10TT under different MNAR assumptions for missing quality-of-life data

Scenario number	MNAR rescaling parameters^a^		Incremental cost^b^ (£) [95% CI]	Incremental QALYs [95% CI]	INMB^c^ (£) [95% CI]	Probability cost-effective^c^ (%)
	*c*_control_	*c*_10TT_				
1 (MAR)	1	1	− 35 [− 504 to 434]	− 0.004 [− 0.074 to 0.066]	− 49 [− 1632 to 1534]	48
2	1	0.95	− 35 [− 504 to 434]	− 0.037 [− 0.107 to 0.032]	− 713 [− 2280 to 853]	19
3	0.95	1	− 35 [− 504 to 434]	0.026 [− 0.044 to 0.095]	550 [− 1022 to 2121]	75
4	0.95	0.95	− 35 [− 504 to 434]	−0.008 [− 0.076 to 0.061]	− 115 [− 1670 to 1440]	44
5	0.95	0.90	− 35 [− 504 to 434]	− 0.041 [− 0.109 to 0.027]	− 780 [− 2321 to 762]	16
6	0.90	0.95	− 35 [− 504 to 434]	0.022 [− 0.046 to 0.091]	484 [− 1063 to 2030]	73
7	0.90	0.90	− 35 [− 504 to 434]	− 0.011 [− 0.078 to 0.057]	− 181 [− 1714 to 1352]	41

All results are based on imputed data and comparing the 10TT arm to the control arm (*n* = 537). For participants with complete cost and effectiveness data (*n* = 166; 31%), the observed incremental cost was − £65 [95% CI − 924 to 794], incremental QALYs was − 0.040 [− 0.169 to 0.088], INMB was − £741 [− 3645 to 2163], and probability cost-effective was 31%

*CI* confidence interval, *INMB* incremental net monetary benefit, *MAR* missing at random, *MNAR* missing not at random, *QALY* quality-adjusted life year, *10TT* Ten Top Tips

^a^How missing quality-of-life data are assumed to differ from the MAR-imputed values. *c*_control_ = 0.9 means that all imputed quality-of-life values in the control arm have been reduced by 10%

^b^Missing costs assumed to be MAR in all scenarios

^c^At a cost-effectiveness threshold of £20,000/QALY

#### Implementation of the Analysis in Stata

The annotated Stata code to conduct the analysis is provided in Online Appendix 2 (see the ESM), and the dataset is described in Online Appendix 1.

*Step 1. Performing Multiple Imputation*

The first step of the analysis is to conduct standard MI (under an MAR assumption), to ‘fill in’ the variables with missing data. The missing HRQoL at each time point and total costs were imputed stratified by arm, using a linear model based on each other, and baseline characteristics (age, sex, study centre, weight, body mass index and baseline HRQoL). We conducted MI by chained equations, using predictive-mean matching, and created 50 imputations. Note that alternative MI approaches, for example, linear regression, would not affect the proposed sensitivity analysis strategy. More detailed guidance on conducting MI in Stata is provided elsewhere [3, 13, 49].

*Step 2. Modifying Imputed Data*

To obtain the imputed data under MNAR, we simply need to multiply each MAR-imputed value by *c*. For example: 

will multiply the imputed values of qol_3 in the control arm by 0.9.

Different versions of the modification could be implemented at this stage (see Sect. 4.2), for example, by alternatively considering an ‘offset’ additive parameter *d*: 

This can be done in turn for each of the scenarios, or storing each of the scenario parameters in a table (matrix) allows Stata to execute this in one step, using a loop. The modified data can then be saved in a single large dataset to facilitate the remaining steps.

*Step 3. Analysing the MNAR Dataset*

The CEA analysis is then applied as usual to each of the MNAR multiply-imputed datasets. To estimate the incremental costs, QALYs and net monetary benefit and their 95% CIs, we have used the ‘mi estimate’ command, which fits the analysis model on each of the imputed datasets, then combines the results using Rubin’s rules [15]. We have also used a non-parametric bootstrap approach to produce the cost-effectiveness plane and the CEAC, with the implementation described in Online Appendix 2 (see the ESM). Further guidance on the analysis of multiply-imputed cost-effectiveness data can be found elsewhere [1, 3, 4, 12].

*Step 4. Reporting*

**Fig. 3:**
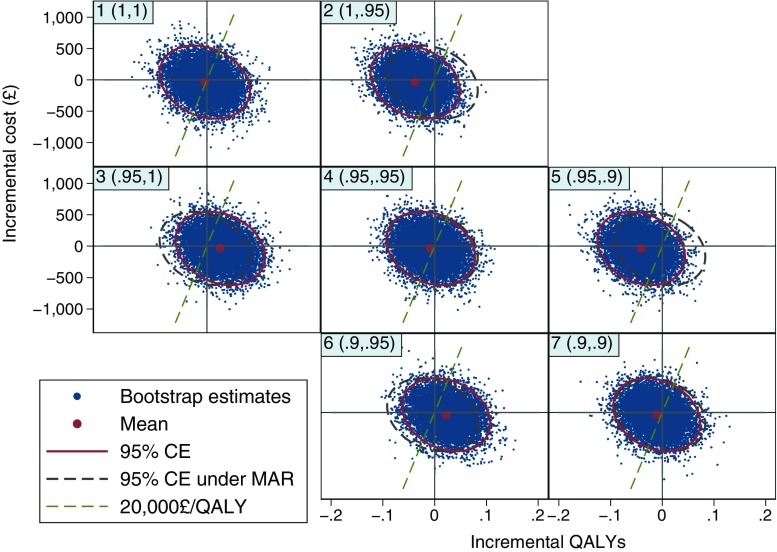
Cost-effectiveness planes under different MNAR assumptions. Headings in top-left corner indicate the scenario number and the MNAR rescaling parameters (*c*_control_, *c*_10TT_). For example, (0.9, 0.9): imputed quality-of-life values have been reduced by 10% in both arms. Each plane is based on 10,000 bootstrap replicates, from 50 imputed datasets. 95% CEs are shown (solid ellipse), alongside the 95% CEs under MAR (scenario 1) as a reference (dashed ellipse). Dashed lines indicate the cost-effectiveness threshold of £20,000 per QALY. *CE* confidence ellipse, *MAR* missing at random, *MNAR* missing not at random, *QALY* quality-adjusted life year, *10TT* Ten Top Tips

**Fig. 4:**
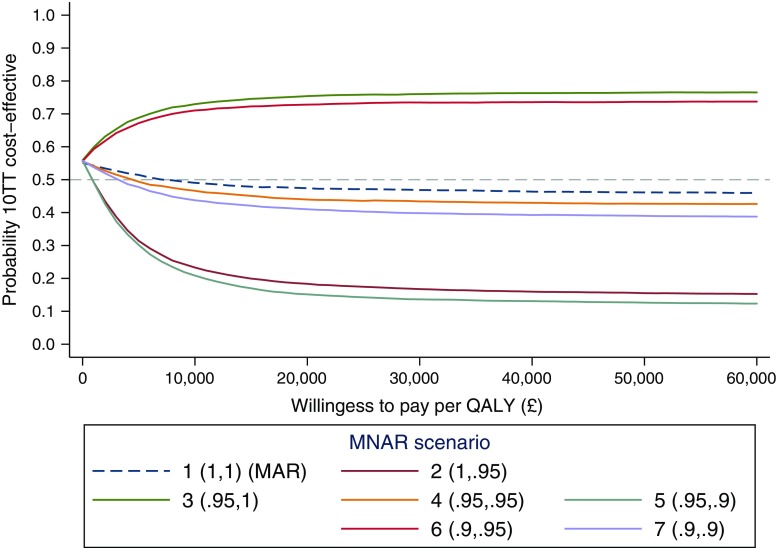
Cost-effectiveness acceptability curves under different MNAR assumptions. Legend indicates the scenario number and the MNAR rescaling parameters (*c*_control_, *c*_10TT_). For example, (0.9,0.9): imputed quality-of-life values have been reduced by 10% in both arms. *MAR* missing at random, *MNAR* missing not at random, *QALY* quality-adjusted life year, *10TT* Ten Top Tips

Clear reporting of the sensitivity analysis results is key to ensure their implications are well understood. We recommend a table which presents the summary findings for each scenario (Table 1). Figure 3, which plots the cost-effectiveness plane for the different MNAR scenarios is also useful to understand the effect of each MNAR assumption, as discussed in the next section. Our discussions with stakeholders indicated that the most intuitive way to summarise the findings was probably overlaying CEACs, showing the probability of the intervention being cost-effective at different thresholds, for each MNAR scenario (Fig. 4). Alternative presentations of the sensitivity analysis results are discussed in Sect. 4.5.

#### Results

The 10TT CEA results under the different missing data scenarios are reported in Table 1, Figs. 3 and 4. In particular, the CEAC (Fig. 4) shows that the probability of 10TT being cost-effective remains relatively stable when MAR departures are assumed to be the same across randomised arms (scenarios 1, 4 and 7). This is also seen in Table 1, where the alternative departures from MAR had little effect on the incremental QALYs in these scenarios. This will usually be the case when the missing data pattern is broadly similar across treatment arms, as the MNAR bias applies roughly equally to each arm and cancels out in the treatment comparison.

As we move through the other scenarios, however, 10TT alternates between being cost-effective and not depending on which arm is assumed to have a stronger MNAR mechanism. For example, 10TT appear unlikely to be cost-effective when we assumed stronger MNAR (lower *c*) for the treatment arm, with a probability of being cost-effective around 0.2 at £20,000 per QALY. Table 1 also shows how the incremental QALYs vary across the different scenarios, while the width of the 95% CI remains relatively similar. Since the magnitude of the incremental QALYs was relatively small, different missing data mechanisms across arms led to substantially different incremental QALYs estimates.

The impact of the different MNAR assumptions can also be readily described in the cost-effectiveness plane (Fig. 3). On the diagonal, where the MAR departures are assumed to be the same in both arms, the joint distribution of incremental QALYs and cost remains relatively unchanged. However, differential changes of the sensitivity parameter (*c*) between arms lead to a shift in the distribution of incremental QALYs to the right (10TT more cost-effective) or left (10TT less cost-effective). These shifts essentially reflect the impact of the MAR departures on the incremental QALYs seen in Table 1. For example, for scenarios where *c* is lower (stronger departure from MAR) in the treatment arm (upper-right off-diagonal plots), the joint distribution is shifted to the left and the proportion of points below the cost-effectiveness threshold (£20,000 per QALY) is lower (10TT less likely to be cost-effective).

## Extensions

Section 3 provided a relatively simple example of a sensitivity analysis. In this section, we discuss possible extensions and further issues around their implementation in practice.

### Missing Cost

In our base-case example, we considered departures from the MAR assumption for the effectiveness endpoint (HRQoL) only. However, it is possible to consider MNAR sensitivity analysis for the cost data as well, following a similar approach.

Table 2 presents the results of a sensitivity analysis for 10TT when both the missing cost and HRQoL data were considered to be MNAR. This involves four parameters, capturing the MAR departure in total costs and HRQoL, in each arm. The missing costs were assumed to be somewhere between MAR and up to 10% higher than observed (i.e. participants who dropped out may have higher health care use). Table 2 suggests that the departures from MAR for the cost endpoint would only have a marginal effect on the overall results, while departures for the HRQoL endpoint can strongly affect the conclusions, particularly if the missing data mechanisms differ between arms. More details on the analysis and the Stata code are provided in Online Appendix 3 (see the ESM).

**Table 2:** Cost-effectiveness of 10TT under different MNAR assumptions for missing cost and effectiveness quality-of-life data

Scenario description	Incremental cost (£) [95% CI]	Incremental QALYs [95% CI]	INMB^a^ (£) [95% CI]	Probability cost-effective^a^ (%)
MAR	− 35 [− 504 to 434]	− 0.004 [− 0.074 to 0.066]	− 49 [− 1632 to 1534]	48
Same MNAR parameters^b^ in the two arms				
− 10% QoL in both arms	− 35 [− 504 to 434]	− 0.011 [− 0.078 to 0.057]	− 181 [− 1714 to 1352]	41
+ 10% cost in both arms	− 25 [− 512 to 462]	− 0.004 [− 0.074 to 0.066]	− 59 [− 1650 to 1532]	47
− 10% QoL and + 10% cost	− 25 [− 512 to 462]	− 0.011 [− 0.078 to 0.057]	− 191 [− 1733 to 1350]	40
Different MNAR parameters^b^ in the two arms				
− 10% QoL in intervention arm	− 35 [− 504 to 434]	− 0.071 [− 0.139 to − 0.002]	− 1378 [− 2932 to 176]	4
− 10% QoL in control arm	− 35 [− 504 to 434]	0.056 [− 0.014 to 0.125]	1148 [− 415 to 2711]	93
+ 10% cost in intervention arm	20 [− 459 to 499]	− 0.004 [− 0.074 to 0.066]	− 104 [− 1691 to 1483]	45
+ 10% cost in control arm	− 80 [− 558 to 398]	− 0.004 [− 0.074 to 0.066]	− 4 [− 1591 to 1583]	50

All results are based on imputed data and comparing the 10TT arm to the control arm (*n* = 537)

*CI* confidence interval, *INMB* incremental net monetary benefit, *MAR* missing at random, *MNAR* missing not at random, *QALY* quality-adjusted life year, *QoL* quality of life, *10TT* Ten Top Tips

^a^At a cost-effectiveness threshold of £20,000/QALY

^b^How missing cost and QoL data are assumed to differ from MAR-imputed values

As the number of variables increase, so does the number of sensitivity parameters, whose values we have to specify. The number of plausible combinations of these parameters can quickly become overwhelming, and it may be best to focus on a limited number of scenarios, or on the parameters that affect the results the most, to allow for a meaningful interpretation.

### Alternative MNAR Parametrisation

In our example, we have rescaled the MAR-imputed HRQoL by a multiplicative factor. As discussed in Sect. 2.3, another popular pattern-mixture approach is to ‘offset’ the data by an additive factor. This is commonly used for continuous outcomes measured on a readily interpretable scale, such as EQ-5D, which is anchored at 0 (death) and 1 (full health). However, for cost data, a multiplicative reduction may be more intuitive; for example, a ‘10% reduction’ may be more readily understood than a ‘£200 reduction’ as the latter is context specific. A multiplicative transformation may therefore be more appealing in the CEA context.

The values of the MNAR parameters could also be varied according to other factors. With longitudinal data, the departure from MAR can be assumed constant over time—as was considered here—or changing over time, for example, with the parameter increasing with time since withdrawal [31, 37]. The parameter can also be applied at different levels of data aggregation, for example, assuming only one of the resource use components is likely to be MNAR. Different parameters could also be used according to the reasons for discontinuing the trial.

In principle, pattern-mixture models are very flexible and the distribution of unobserved data could take any shape or form. While it can be tempting to consider more complex models (e.g. additional parameters), it can make elicitation and interpretation challenging. In our view, simple offsets or rescaling of the MAR distribution (allowed to differ by arm) should usually provide sufficient span for a comprehensive sensitivity analysis, while remaining sufficiently transparent.

### Choosing the MNAR Parameters

One of the main concerns about conducting an MNAR analysis is how to choose plausible sensitivity parameter values. Several approaches and sources of information can be used for this purpose. One potential approach is to formally elicit ‘experts’ beliefs on the missing data distribution [28]. These `experts’ can be anyone who can contribute knowledge in understanding the missing data, such as trial investigators, clinicians, or patients. Mason et al. have developed a useful framework for eliciting expert opinion about MNAR mechanisms in CEA [36]. The experts’ beliefs, capturing the most likely value for the MNAR parameters, and the uncertainty in that value, can then be incorporated into the analysis model (see Sect. 4.4).

Alternatively, one could simply use a ‘tipping point’ or threshold analysis approach. This involves changing the MNAR parameter until a different conclusion is reached (for example, being or not being cost-effective). The analyst can then discuss with the relevant experts the plausibility of this value. This approach is appealing because it is more readily implemented and less time-consuming than formal elicitation, and may provide sufficient information for the decision problem at hand, especially when results are robust to a wide range of assumptions. However, what constitutes a ‘change of conclusion’ may not be uniquely defined, and it may be difficult to implement with multiple sensitivity parameters.

An intermediate approach would be to agree on plausible sensitivity scenarios with those involved in the trial or regulators, for example, at a steering committee meeting. A ‘most likely’ scenario and several ‘most extreme’ scenarios could be agreed on, without formally eliciting the uncertainty in the parameters. The scenarios should cover all plausible situations, so that readers can be confident that missing data are unlikely to affect the CEA conclusions beyond what is reported in the sensitivity analysis.

Analysts should also consider how missing data are addressed in the trial primary (clinical) analysis, and the elicitation could be done jointly when suitable. The elicitation should ideally be conducted around the final stages of data collection and be ‘pre-specified’ before the trial results are known.

Overall, a clear understanding of the reasons for missing data in the specific trial context, discussions with relevant ‘experts’, and insights drawn from the literature are key to inform the choice of sensitivity parameters.

### Probabilistic Parameters

An alternative to reporting results for specific sensitivity parameters values is to incorporate the uncertainty around the parameters into the analysis model. This is a natural approach when a formal elicitation of the parameter’s value and its uncertainty has been conducted (Sect. 4.3). While the analysis can be conducted using a Bayesian framework [36], it can also be implemented using MI [28, 37]. To do so, instead of rescaling all the imputed dataset by a fixed value, a random parameter value is drawn from the elicited distribution for each of the imputed datasets. An example is provided in Online Appendix 4 (see the ESM).

This probabilistic approach is particularly appealing as it incorporates the uncertainty related to MNAR into the analytical model, providing a ‘single’ answer. It can be particularly relevant, for example, if the result is to be incorporated in a larger decision model.

However, some stakeholders found this approach less comprehensive than the reporting under different MNAR scenarios. Indeed, this approach also relies on making a single assumption (that the uncertainty was captured appropriately), whereas a range of plausible scenarios may be more readily interpretable in showing how different missing data mechanisms could result in different conclusions.

### Presentation of Results

We have shown how to report the results for different MNAR scenarios by displaying the resulting CEACs. This was flagged by stakeholders as an accessible way to report the results, but they have also recognised that alternative graphical representations may be preferred depending on the decision problem at hand. In this section, we illustrate some of these graphical tools (Stata code provided in Online Appendix 5; see the ESM).

**Fig. 5:**
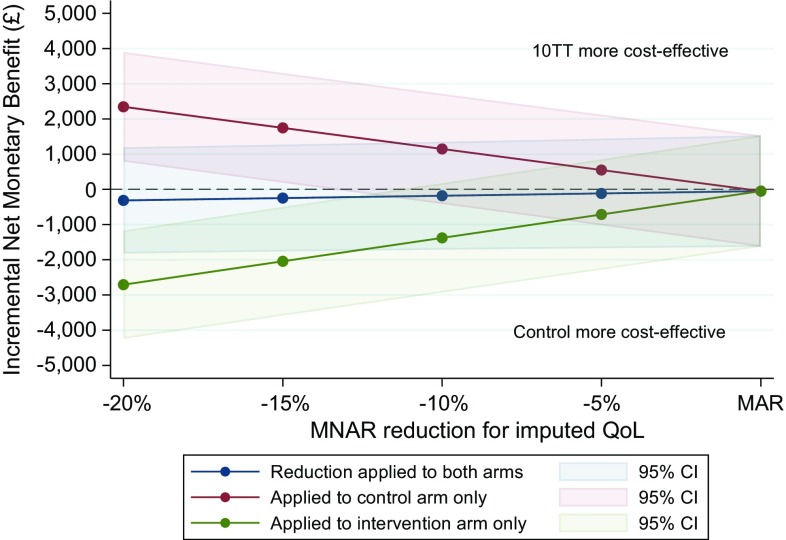
Alternative presentation: incremental net monetary benefit of 10TT compared to control arm (at £20,000/QALY), for different values of the MNAR rescaling parameter. *CI* confidence interval, *MAR* missing at random, *MNAR* missing not at random, *QoL* quality of life, *10TT* Ten Top Tips

For example, Fig. 5 shows the INMB (and CIs) for values of the *c* parameter, ranging from 0.8 to 1. The parameter is applied to both arms simultaneously, or only one of the arms.

**Fig. 6:**
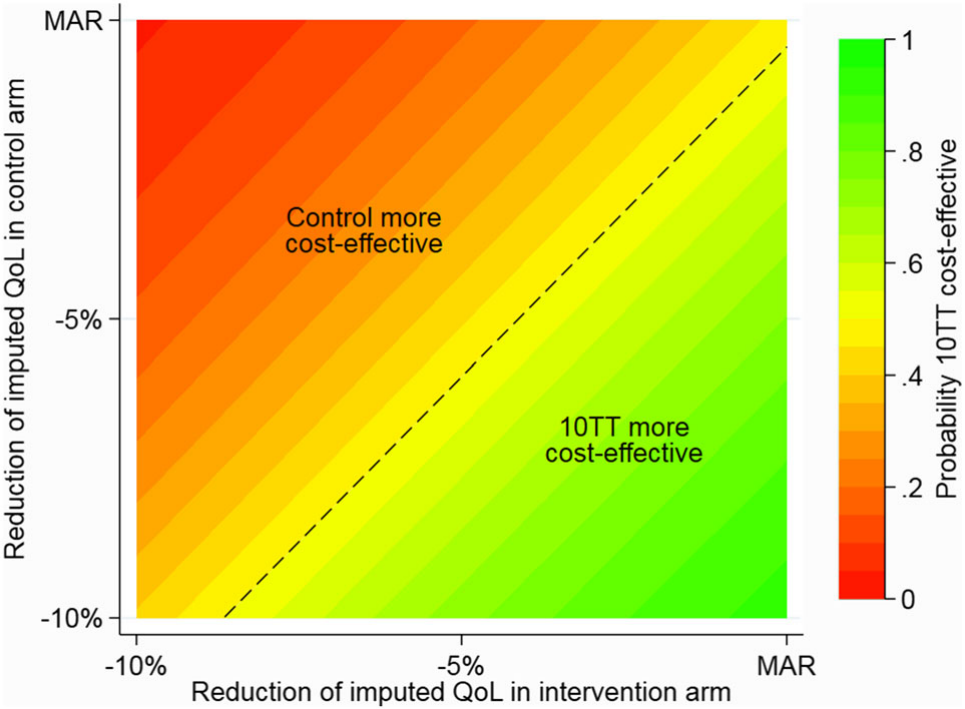
Alternative presentation: contour plot of the probability of 10TT being more cost-effective than control (at £20,000/QALY), for different values of MNAR rescaling parameters in the control and intervention arms. *MAR* missing at random, *MNAR* missing not at random, *QALY* quality-adjusted life year, *QoL* quality of life, *10TT* Ten Top Tips

Alternatively, a more comprehensive description of possible combinations of the sensitivity parameters across treatment arms is plotted in Fig. 6. This ‘colour-coded graph’ (or contour plot) provides a useful tool to interpret the implications of different departures from MAR on the overall decision. For example, it illustrates that for lower values of *c* (stronger departure from MAR) in the intervention arm compared to the control group, the 10TT intervention is unlikely to be cost-effective (red/orange area).

## Discussion

In this tutorial, we have outlined different approaches for conducting sensitivity analysis for missing data in CEA. We focused on one particularly accessible approach, based on pattern-mixture modelling with MI, and illustrated how it can be implemented in practice. While this is not, in any sense, the final word, we believe that more widespread use of the approach described here would represent a substantial step towards realising the regulatory call for sensitivity analysis.

As Sect. 2 highlights, numerous approaches to MNAR analyses are possible, and there is a large literature on this topic [11, 18, 37]. However, we believe the approach illustrated here has the key advantages of accessibility, flexibility, and transparency. Transparency is indeed the principal requirement for these sensitivity analyses to serve their purpose, as the plausibility of their underlying assumptions needs to be clearly understood and critically assessed by a broad readership [2, 16, 31]. The straightforward implementation of the analysis within an MI framework makes it accessible to the increasing number of analysts who are routinely using MI. It can also be readily implemented within any statistical software with MI (Stata, R, SAS, SPSS, etc.).

Ready implementation allows the focus to be on identifying relevant MNAR scenarios and assessing their plausibility. We discussed here several approaches that can be used in practice, whose suitability will depend on each situation. Some approaches are more rigorous, but more time-consuming, while others are cruder, but still informative. Deciding on the relevant scenarios is likely to involve discussion with other collaborators, and the analysts should be able to explain the different assumptions in non-technical language. Another challenge is the reporting of the results: how can the analyst ensure that the sensitivity analysis is comprehensive, without being overwhelming for the readers? We have suggested a framework where the analysis is conducted under a limited number of plausible scenarios, and the results reported in a table and on a combined CEAC, but also discussed alternative presentations.

The proposed framework is not without some limitations, however. First, every trial raises different issues, and it is not possible to recommend a universal framework for MNAR sensitivity analyses. The framework suggested here is nevertheless relatively flexible, and should be suitable in a wide range of settings, including longitudinal and cluster-randomised trials. Secondly, an assumption such as ‘the missing HRQoL are 10% lower’ could be too simplistic to capture the varied reasons behind missing data. However, it is important to consider this in light of several aspects. We are primarily interested here, as is usually the case in randomised trials, in estimating mean differences between groups. To obtain valid conclusions, it is therefore not necessary to predict accurately each missing value, but only the *average* difference between observed and missing data. Also, the true missing data mechanism is always unknown, and the aim of the sensitivity analysis is not to provide a definitive answer, but to indicate how conclusions could differ under different missing data assumptions. Finally, the framework proposed here was for continuous outcomes such as cost and quality of life. While the main ideas of the framework are relevant for other outcomes (e.g. binary or survival), they do raise additional challenges, especially around model compatibility and elicitation [37]. For example, differences between observed and missing data in terms of ‘odds ratios’ may be more difficult to elicit and interpret.

While this tutorial focuses on within-trial CEA, a similar sensitivity analysis approach could possibly be used in observational settings, for example, when analysing routinely collected data, where the issue of informative missing data may arguably be even more important.

This tutorial highlights several areas where further research could improve the value of CEA for decision making in the presence of missing data. A particularly interesting alternative MNAR approach is ‘reference-based’ or ‘controlled’ imputation, where the missing data are assumed to follow a distribution that is ‘borrowed’ from another group. For example, in a trial comparing a drug to placebo, it could be assumed that patients dropping out from the experimental arm have stopped taking their treatment, and therefore follow a similar pattern to that seen in the control arm [33]. This approach is appealing as it sidesteps the elicitation of quantitative parameters required for selection or pattern-mixture models, and instead formulates the MNAR assumption in a qualitative way. It was well received when discussed with stakeholders, but, to our knowledge, has not yet been used in the CEA context. Relevant areas for further research also include incorporating the sensitivity analysis results into broader decision models and, related to this, conducting sensitivity analysis without patient-level data. One possibility could be to approximate the MNAR bias based on the proportion of missing data, and to retain the analysis standard errors as a measure of sampling uncertainty. Further guidance on how to best address missing binary and survival endpoints is still needed. While we propose some routes for eliciting sensitivity parameters, this critical aspect deserves further attention, and is likely to evolve as MNAR analyses become more routinely performed.

In summary, CEA based on incomplete data should routinely assess whether the study’s conclusions are robust to potential departures from the standard MAR assumption. This paper described some approaches to conducting these sensitivity analyses, and illustrated the application of a practical, accessible framework using pattern-mixture models with MI. This approach builds on the increasing use of MI in CEA and should provide an important step towards improving practice in trial-based CEA.

## Electronic supplementary material

Below is the link to the electronic supplementary material.

Supplementary material 1 (PDF 263 kb)
