# Correction to: Sensitivity Analysis for Not-at-Random Missing Data in Trial-Based Cost-Effectiveness Analysis: A Tutorial

**DOI:** 10.1007/s40273-019-00776-7

**Published:** 2019-03-09

**Authors:** Baptiste Leurent, Manuel Gomes, Rita Faria, Stephen Morris, Richard Grieve, James R. Carpenter

**Affiliations:** 1 Department of Medical Statistics, London School of Hygiene and Tropical Medicine, Keppel Street, London, WC1E 7HT UK; 2 Department of Applied Health Research, University College London, London, UK; 3 Centre for Health Economics, University of York, York, UK; 4 Department of Health Services Research and Policy, London School of Hygiene and Tropical Medicine, London, UK; 5 MRC Clinical Trials Unit at University College London, London, UK

## Correction to: PharmacoEconomics (2018) 36:889–901 10.1007/s40273-018-0650-5

The Open Access license, which previously read:

Open Access This article is distributed under the terms of the Creative Commons Attribution-NonCommercial 4.0International License (http://creativecommons.org/licenses/by-nc/4.0/), which permits any noncommercial use, distribution,and reproduction in any medium, provided you give appropriate credit to the original author(s) and the source, provide a link to the Creative Commons license, and indicate if changes were made.

Should read:

Open Access This article is distributed under the terms of the Creative Commons Attribution 4.0 International License (http://creativecommons.org/licenses/by/4.0/), which permits unrestricted use, distribution, and reproduction in any medium, provided you give appropriate credit to the original author(s) and the source, provide a link to the Creative Commons license, and indicate if changes were made.

The original article was corrected.

