# Supplementary material for: Sensitivity Analysis for Not-at-Random Missing Data in Trial-Based Cost-Effectiveness Analysis: A Tutorial
Source: Pharmacoeconomics. 2018 Apr 20;36(8):889–901. doi: 10.1007/s40273-018-0650-5 (PMC6021473; doi:10.1007/s40273-018-0650-5)
Supplement: Supplementary file 1 — Supplementary material 1 (PDF 263 kb) [file 40273_2018_650_MOESM1_ESM.pdf]

Online Appendix 1 – Ten Top Tips data description

Variable	Description	N	Missing (n)	Missing (%)	Min	Max	Mean	Standard Deviation
**Baseline data**								
`pid`	Participant code	537	0	0	1	537	-	-
`site_code`	Practice code	537	0	0	1	14	-	-
`arm`	Trial arm	537	0	0	0	1	-	-
`sex`	Gender	537	0	0	1	2	-	-
`age`	Age (years)	537	0	0	18	83	56.80	12.73
`weight_0`	Weight at baseline (kg)	537	0	0	70	177	100.83	17.20
`bmi_0`	BMI at baseline (kg/m^2)	537	0	0	30	61	36.38	5.10
`qol_0`	HRQoL at baseline	537	0	0	-.18	1	0.75	0.25
**Quality of life**								
`qol_3`	HRQoL – 3 months	395	142	26.4	-.18	1	0.77	0.25
`qol_6`	HRQoL – 6 months	322	215	40	-.07	1	0.77	0.25
`qol_12`	HRQoL – 12 months	286	251	46.7	-.18	1	0.77	0.24
`qol_18`	HRQoL – 18 months	259	278	51.8	-.26	1	0.75	0.25
`qol_24`	HRQoL – 24 months	284	253	47.1	-.14	1	0.77	0.24
`qaly`	QALYs	166	371	69.1	-.1	2	1.50	0.42
**Cost**								
`totalcost`	Total costs 0-24 months (£)	393	144	26.8	23	27578	1997.81	2546.65

HRQoL= Health-related quality of life (derived from EQ-5D questionnaire), QALYs = Quality Adjusted Life Years, BMI = Body mass index
Notes: missing data at baseline were mean-imputed. Data stored in a ‘wide’ format, with one record per participant.

Online Appendix 2 – MNAR sensitivity analysis of the 10TT trial – Stata code

Step 1 - Perform multiple imputation under MAR

```
   use "10TT_CEA_tutorial.dta", clear

   *Generate missing data indicators
      //Needed for step 2
      misstable sum  qol_3-qol_24 totalcost qaly ,  gen(miss_)

   *Set imputation
      mi set flong
      mi register imputed qol_3-qol_24 totalcost
      mi register passive qaly

   *Perform imputation
      mi impute chained (pmm, knn(10)) qol_3-qol_24 totalcost = i.sex age i.site_code weight_0
bmi_0 qol_0, add(50) by(arm) rseed(123456)
      //Multiple imputation by chained equations, using predictive mean matching.
      //Imputing the Qol at each follow up and the total cost, using baseline variables.
      //Performing 50 imputations, stratified by arm.
      //rseed()is for reproducibility.
      //Alternatively could use "mi impute chained (regress)" or "mi impute mvn"

   *Calculate QALY
      mi passive: replace qaly=0.125*qol_0 + 0.25*qol_3 + 0.375*qol_6 + (0.25*1.965)*qol_12 +
(0.5*0.965)*qol_18 + (0.25*0.965)*qol_24
      //Area under the curve of the individual QoL (discounted by 3.5% the 2nd year)
      //'mi passive' replace QALY only in imputed datasets.

   *Save imputed dataset
      save "10TT_MI.dta", replace
```

Step 2 - Modifying imputed data

```
   ** Define MNAR scenarios of interest
      //MNAR parameters values are stored in a matrix
      matrix mnar_param = ( 1.0,1.0 \ 1.0,0.95 \ 0.95,1.0 \ 0.95,0.95 \ 0.95,0.9 \ 0.9,0.95 \
0.9,0.9 )
      matrix colnames mnar_param = C_ctr C_int
      matrix list mnar_param
      global nscen = rowsof(mnar_param)  // Saving number of scenarios in global macro

   ** Modify MI data
      clear
      save "10TT_MI_MNAR.dta", replace emptyok //Empty dataset to start with
      forvalues s = 1/$nscen {     //Loop over each MNAR scenarios
            use "10TT_MI.dta" , clear

            *Save scenario info
               gen scenid=`s'
               local q0 = mnar_param[`s',1]
               local q1 = mnar_param[`s',2]
               gen scenario= "`s' (`q0',`q1') "

            *Modify QoL values
               foreach var of varlist qol_3 qol_6 qol_12 qol_18 qol_24 {
                  replace `var'=`var'*mnar_param[`s',1] if miss_`var'==1 & arm==0
                  replace `var'=`var'*mnar_param[`s',2] if miss_`var'==1 & arm==1
                  }

            *Calculate modified QALY
               mi passive: replace qaly=0.125*qol_0 + 0.25*qol_3 + 0.375*qol_6 +
(0.25*1.965)*qol_12 + (0.5*0.965)*qol_18 + (0.25*0.965)*qol_24

            *Append and save
               // The results for all MNAR scenarios are appended in a large dataset to
facilitate remaining steps.
               append using "10TT_MI_MNAR.dta"
               save  "10TT_MI_MNAR.dta", replace
            }
```

Step 3a - Analysis: Incremental cost, effect, ICER and INMB, using Rubin's rules.

```
*** Incremental cost and effect
   *Cost:
      //Have not been MNAR-modified, same for all scenarios.
      use "10TT_MI_MNAR.dta" if scenid==1, clear
      mi estimate: mean totalcost if arm==0
      mi estimate: mean totalcost if arm==1
      mi estimate: regress totalcost arm

   *QALY:
      forvalues s = 1/$nscen {
         use "10TT_MI_MNAR.dta" if scenid==`s', clear
         list scenario in 1
         mi estimate: mean qaly if arm==0
         mi estimate: mean qaly if arm==1
         mi estimate: regress qaly arm
         }

*** ICER
   forvalues s = 1/$nscen {
      use "10TT_MI_MNAR.dta" if scenid==`s', clear
      list scenario in 1
      *Incremental cost
         mi estimate: regress totalcost arm
         local incc= el(e(b_mi),1,1)
      *QALY
         mi estimate: regress qaly arm
         local incq= el(e(b_mi),1,1)
      *Display ICER
         list scenario in 1
         display "MNAR scenario `s' : ICER = "  `incc'/`incq'
      }

*** NMB
   forvalues s = 1/$nscen {
      use "10TT_MI_MNAR.dta" if scenid==`s', clear
      list scenario in 1
      gen inb20=qaly*20000-totalcost
      mi estimate: regress inb20 arm
      }

*** Probability cost effective
   forvalues s = 1/$nscen {
      use "10TT_MI_MNAR.dta" if scenid==`s', clear
      list scenario in 1
      gen inb20=qaly*20000-totalcost
      mi estimate: regress inb20 arm
      local pce = normal(el(r(table),1,1)/el(r(table),2,1) )
      display "Probabiliy cost effective = " `pce'
      }
```

Step 3b - Analysis: CEP and CEAC plots, using non-parametric bootstrap

```
*** Bootstrap
   //Conduct bootstrap re-sampling on imputed dataset
   //Note that alternatives to bootstrap could have been considered here (cf. Faria et al.)

   ** Set up
      *Program returning incremental cost and effect
         capture program drop ceestim
         program define ceestim , rclass
            regress qaly arm
            return scalar inc_qaly = _b[arm]
            regress totalcost arm
            return scalar inc_cost = _b[arm]
            end
      *Dataset to store BS estimates
         clear
         save "bootstrap_mnar.dta", replace emptyok //Empty dataset

   ** Run bootstrap
      //Note: different approaches have been suggested to combine MI and BS
      //(cf. Schomaker and Heumann, arXiv:1602.07933)
```

```
         //Here we are using one possible approach: drawing bootstrap samples from each of the
imputed dataset separately, then pooling the estimates.
         forvalues s = 1/$nscen {
            forvalues m = 1/50 {
               use "10TT_MI_MNAR.dta" if scenid==`s' & _mi_m==`m', clear
               //Open one MI dataset, for one MNAR scenario.
               bootstrap inc_cost=r(inc_cost) inc_qaly=r(inc_qaly), ///
                       reps(200) strata(arm) saving("bsres.dta", replace) : ceestim
                     // Bootstrapping the incremental cost and effect.
                     // 200 BS replications for each imputed dataset, stratified by arm.
               *Pool all estimates
                  use "bsres.dta", clear
                  gen _mi_m=`m'
                  gen scenid=`s'
                  append using "bootstrap_mnar.dta"
                  save "bootstrap_mnar.dta", replace
               }
            }

      ** Clean bootstrap dataset
         *Add scenario label (used for graphs)
            use "10TT_MI_MNAR.dta", clear
            keep scenid scenario
            duplicates drop
            merge 1:m scenid using "bootstrap_mnar.dta", nogenerate
         *Sort and save
            sort scenid _mi_m
            compress
            save "bootstrap_mnar.dta", replace


   *** Cost-effectiveness plane
      use "bootstrap_mnar.dta" , clear
      bysort scenario: egen meanc=mean(inc_cost)
      bysort scenario: egen meanq=mean(inc_qaly)

      *Graph
         graph twoway scatter inc_cost inc_qaly, msize(*0.1) || scatter meanc meanq, ///
                     by(scenario, holes(3 7) compact leg(off)) ///
                     xlab(-0.2(0.1)0.2) xtitle("Incremental QALY") ///
                     ylab(-1000(500)1000,  nogrid angle(horizontal)) ///
                     ytitle("Incremental Cost (£)") ///
                     yli(0,lc(black) lw(thin))  xli(0,lc(black) lw(thin)) ///
                     name(CEP, replace)


   *** Cost-Effectiveness Acceptability Curve

      *Calculate probability cost effective at different threshold.
         postfile ceac scenid str12 scenario wtp proba using "ceac.dta", replace
         // Set-up 'postfile' to store results
         forvalues s = 1/$nscen {
            use "bootstrap_mnar.dta" if scenid==`s', clear
            forvalues wtp = 0(1000)60000 {
               qui: count if (inc_qaly*`wtp'-inc_cost)>0
               local p = `r(N)' / _N  //Proportion of cost-effective BS replicates
               post ceac (scenid[1]) (scenario[1])  (`wtp') (`p')
               }
            }
         postclose ceac //Closing postfile

      *Graph
         use "ceac.dta", clear
         separate proba, by(scenario) veryshortlabel gen(proba_)
            //Create a new variable for each MNAR scenario (needed to show on same graph)
         graph twoway line proba_* wtp, ///
               xlab(0(10000)60000, format(%9.0fc)) ///
               xtitle("Willingess to pay per QALY (£)") ///
               yscale(range(0 1)) ylab(0(0.1)1, nogrid angle(horizontal) format(%2.1f)) ///
               ytitle("Probability 10TT cost effective") ///
               yline(0.5,lc(gs10) lw(thin) lpattern(dash)) ///
               legend(label(1 "1 (1,1) (MAR)") title("MNAR scenario")) rows(3) hole(3 7)) ///
               name(CEAC, replace)
```

Online Appendix 3 – MNAR sensitivity analysis for missing cost and effectiveness

We reanalysed the 10TT trial data, this time considering the missing cost data could also be MNAR.

1) Scenarios of interest

The MNAR scenarios were defined by four parameters:

- c_{Q0} : the MNAR rescaling factor for the imputed quality-of-life score (QoL) in the control group. For example, c_{Q0} =0.9 correspond to reducing MAR-imputed QoL values in the control group by 10%.
- c_{Q1} : rescaling factor for HRQoL in the intervention group
- c_{c0} : rescaling factor for total cost in the intervention group
- c_{c1} : rescaling factor for total cost in the intervention group

We considered eight scenarios, covering a range of MNAR variation for cost and QoL. We considered the missing QoL more likely to be lower, and the cost higher, than under MAR.

Scenario description	MNAR rescaling parameters			
	**QoL in control group**	**QoL in intervention group**	**Cost in control group**	**Cost in intervention group**
1. (MAR)	1	1	1	1
**Same parameters in both arms**				
2. -10% QoL in both arms	-10%	-10%	1	1
3. +10% cost in both arms	1	1	+10%	+10%
4. -10% QoL and +10% cost	-10%	-10%	+10%	+10%
**Different parameters by arm**				
5. -10% QoL in intervention arm	1	-10%	1	1
6. -10% QoL in control arm	-10%	1	1	1
7. +10% cost in intervention arm	1	1	1	+10%
8. +10% cost in control arm	1	1	+10%	1

2) Stata code to transform QoL and cost

```
   ** Define scenarios
      matrix mnar_param = (1.0,1.0,1.0,1.0 \  0.9,0.9,1.0,1.0 \ 1.0,1.0,1.1,1.1 \
0.9,0.9,1.1,1.1 \ 1.0,0.9,1.0,1.0 \ 0.9,1.0,1.0,1.0\ 1.0,1.0,1.0,1.1\ 1.0,1.0,1.1,1.0)
      matrix colnames mnar_param = Q0 Q1 C0 C1
      matrix list mnar_param
      global nscen = rowsof(mnar_param)  // Global macro, number of scenarios.

   ** Modify MI data
      clear
      save "10TT_MI_MNAR_cost.dta", replace emptyok
      forvalues s = 1/$nscen {
            use "10TT_MI.dta" , clear
            *MNAR parameters variable
               gen scenid=`s'
               local q0 = mnar_param[`s',1]
               local q1 = mnar_param[`s',2]
               local c0 = mnar_param[`s',3]
               local c1 = mnar_param[`s',4]
               gen scenario= "`s' (`q0',`q1',`c0',`c1') "
            *Modify QoL values
               foreach var of varlist qol_3 qol_6 qol_12 qol_18 qol_24 {
                  replace `var'=`var'*mnar_param[`s',1] if miss_`var'==1 & _mi_m>0 & arm==0
                  replace `var'=`var'*mnar_param[`s',2] if miss_`var'==1 & _mi_m>0 & arm==1
                  }
            *Modify cost
```

```
            replace totalcost=totalcost*mnar_param[`s',3] if miss_totalcost==1 & _mi_m>0 &
arm==0
            replace totalcost=totalcost*mnar_param[`s',4] if miss_totalcost==1 & _mi_m>0 &
arm==1
         *Calculate new QALY
            mi passive: replace qaly=0.125*qol_0+0.25*qol_3+0.375*qol_6+(0.25*1.965)*qol_12
+(0.5*0.965)*qol_18+(0.25*0.965)*qol_24
         *Append and save
            append using "10TT_MI_MNAR_cost.dta"
            save "10TT_MI_MNAR_cost.dta", replace
      }
```

3) Results

Scenario description	Incremental cost (£) [95% CI]	Incremental QALYs [95% CI]	INMB[a] (£) [95% CI]	Probability cost-effective[a]
1. MAR	-35 [-504 to 434]	-.004 [-.074 to .066]	-49 [-1,632 to 1,534]	48%
**Same MNAR parameters[b] in the two arms**				
2. -10% QoL in both arms	-35 [-504 to 434]	-.011 [-.078 to .057]	-181 [-1,714 to 1,352]	41%
3. +10% cost in both arms	-25 [-512 to 462]	-.004 [-.074 to .066]	-59 [-1,650 to 1,532]	47%
4. -10% QoL and +10% cost	-25 [-512 to 462]	-.011 [-.078 to .057]	-191 [-1,733 to 1,350]	40%
**Different MNAR parameters[b] in the two arms**				
5. -10% QoL in intervention arm	-35 [-504 to 434]	-.071 [-.139 to -.002]	-1378 [-2,932 to 176]	4%
6. -10% QoL in control arm	-35 [-504 to 434]	.056 [-.014 to .125]	1148 [-415 to 2,711]	93%
7. +10% cost in intervention arm	20 [-459 to 499]	-.004 [-.074 to .066]	-104 [-1,691 to 1,483]	45%
8. +10% cost in control arm	-80 [-558 to 398]	-.004 [-.074 to .066]	-4 [-1,591 to 1,583]	50%

MAR missing at random, MNAR missing not at random, QALY quality-adjusted life year, INMB incremental net monetary benefit, QoL quality-of-life, 10TT Ten Top Tips, CI confidence interval

[a] At a cost-effectiveness threshold of £20,000/QALY.

[b] How missing cost and QoL data are assumed to differ from MAR-imputed values.

4) Cost-effectiveness acceptability curve

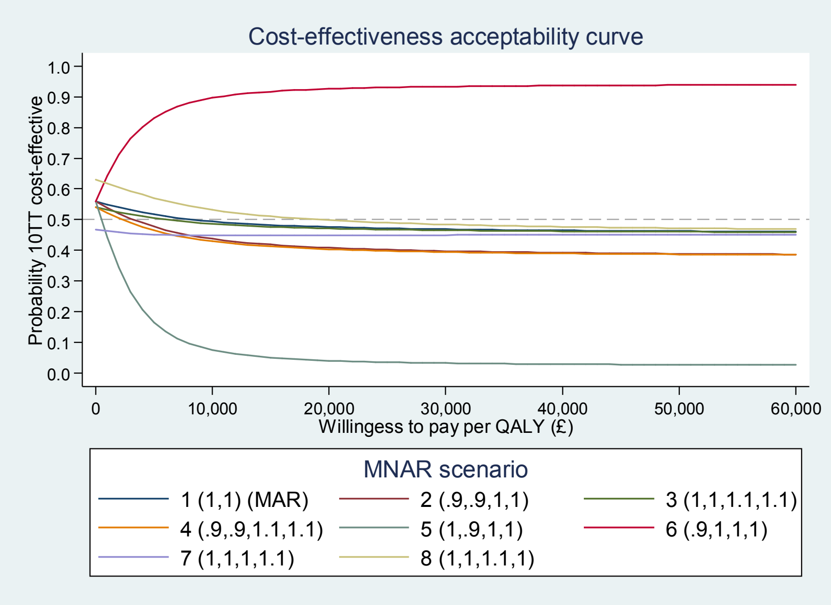

We can see here that a departure from the MAR assumption for the costs is unlikely to affect significantly the findings, even if the missing costs are assumed 10% higher than under MAR only in the intervention arm.

However, departure from the MAR assumption for QoL could importantly affect the conclusions, particularly if the MNAR mechanism is not the same in each arm. The results for varying MNAR parameters for QoL, as reported in the Section 3 of the tutorial, is probably of primary interest in this case.

Online Appendix 4 - Probabilistic MNAR parameters

1) Distribution of the parameters

In this example, let us assume we believe the rescaling parameter *c* to be around 0.95, with a standard deviation of 0.025 (this standard deviation corresponds to being 95% certain that the true parameter value is somewhere between 1 and 0.90). We want to draw two correlated values from that distribution (c_{control} , c_{10TT}).

A correlation would capture how the values of c_{control} and c_{10TT} are related, for example if the departure from MAR is strong in one arm, it could be more likely to also be the case in the other arm (positive correlation). The difficulty of eliciting the correlation parameter has been discussed elsewhere[1,2]. One solution is to simply assume independence. Indeed, this should result in a slightly conservative estimate for the difference between arms (assuming the correlation is usually positive), and the difference will typically be negligible[1,2] (to confirm this, the analysis could also be repeated with different correlations).

We will therefore draw two parameters from the following distribution:

$$\begin{pmatrix} c_{Control} \\ c_{10TT} \end{pmatrix} \sim N\left(\begin{pmatrix} 0.95 \\ 0.95 \end{pmatrix}, 0.025^2 \begin{pmatrix} 1 & 0 \\ 0 & 1 \end{pmatrix}\right)$$

2) Analysis implementation

We incorporated the random draw as part of the multiple imputation procedure, by drawing a different set of (c_{control} , c_{10TT}) for each of the imputed dataset, and rescaling each dataset accordingly. The standard analysis of the multiply imputed datasets (i.e. Rubin's rules) should then take into account of both the imputation and the MNAR uncertainty, and approximate a fully Bayesian analysis[1]. Note that a sufficient number of imputations are needed to perform multiple draws of (c_{control} , c_{10TT}), to obtain sufficiently stable results (negligible Monte Carlo error).

3) Stata code

```
** Define parameters distribution
   matrix C = (1,0.0,1) //Uncorrelated draw
   global mu = 0.95
   global sd = 0.025

** Modify MI data
   //Each MI dataset is MNAR-modified according to parameters drawn from the distribution
   use "10TT_MI.dta", clear

   *Draw random parameters
      set seed 1234  //seed for reproducibility
      drawnorm c0 c1, corr(C) cs(lower) //Draw values from 2 correlated normal distribution
      replace c0 = $mu + $sd *c0  // Transform to wanted mean and SD
      replace c1 = $mu + $sd *c1
      bysort _mi_m: replace c0 = c0[1]  //Same parameter value for each imputed dataset
      bysort _mi_m: replace c1 = c1[1]

   *Modify QoL values
      foreach var of varlist qol_3 qol_6 qol_12 qol_18 qol_24 {
         replace `var'=`var'*c0 if miss_`var'==1 & arm==0
         replace `var'=`var'*c1 if miss_`var'==1 & arm==1
         }
```

[1] White I.R., et al. "Eliciting and using expert opinions about dropout bias in randomized controlled trials." *Clinical Trials* 4.2 (2007): 125-139.
[2] Carpenter J., and Kenward M., "Multiple imputation and its application." John Wiley & Sons, 2012.

```
*Calculate modified QALY
   mi passive: replace qaly=0.125*qol_0 + 0.25*qol_3 + 0.375*qol_6 + (0.25*1.965)*qol_12 +
(0.5*0.965)*qol_18 + (0.25*0.965)*qol_24

*Save
   save "10TT_MI_MNAR_probabilistic.dta", replace
```

4) Results

**Scenario description**	**Incremental cost (£) [95% CI]**	**Incremental QALYs [95% CI]**	**INMB[a] (£) [95% CI]**	**Probability cost-effective[a]**
Probabilistic MNAR parameters	-35<br>[-504 to 434]	-.004<br>[-.085 to .076]	-50<br>[-1816 to 1716]	47.8%

QALY quality-adjusted life-years, INMB incremental net monetary benefit
[a] At a cost-effectiveness threshold of £20,000/QALY.

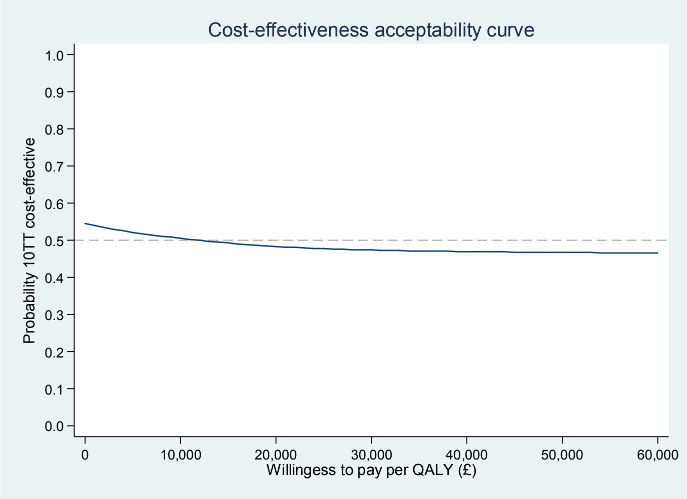

We can see that the resulting incremental estimates are very close to the MAR results. This is likely to be the case when i) the proportion of missing data is relatively similar between arms, and ii) the MNAR parameters have the same mean value in each arm.
However, the probabilistic analysis increases the uncertainty (wider confidence intervals, less steep CEAC), although this is not strongly seen here.
Note that the *within* arm QALYs estimates would differ from those under MAR (not shown here). The rescaling parameter is assumed to be around 0.95, meaning missing QoL are expected to be somewhat lower than under MAR, resulting in lower QALYs (in both arms).

Online Appendix 5 – Presentation of results

1) Net Monetary Benefit over range of MNAR parameter values

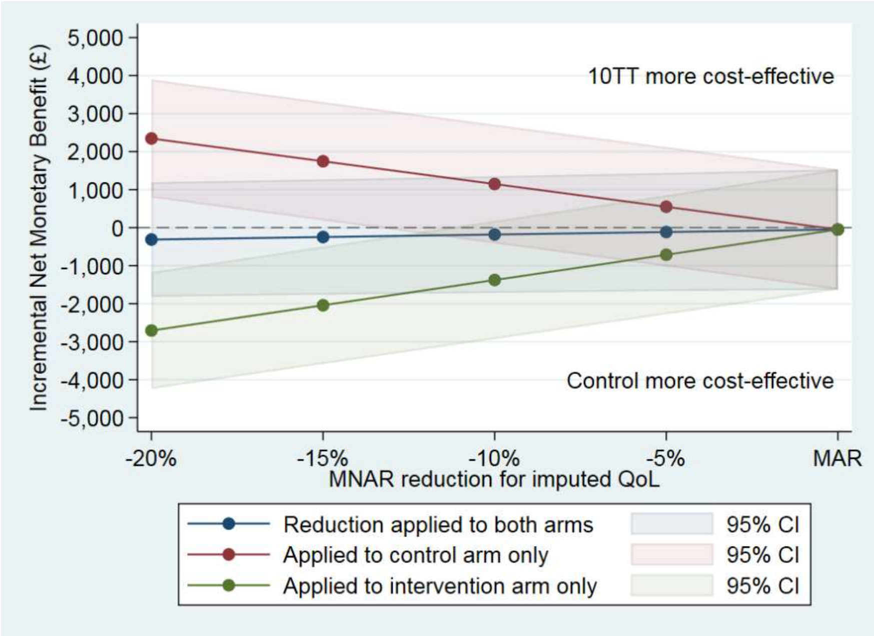

Note: example shown here with INMB, but could also display other results such as ICER or probability of being cost-effective.

<u>Stata code:</u>

```
** Calculate results for different MNAR parameters

postfile inbgraph c c0 c1 armscat inb ll ul using "inbgraph.dta", replace  //Set up 'postfile'
to save results

forvalues c=0.8 0.85 to 1.0 { // Range of MNAR parameter values
forvalues armscat=1/3 {       // 3 series: applying MNAR parameter to either arm, or both
   local c0=cond(`armscat'==1 | `armscat'==2,`c',1)
   local c1=cond(`armscat'==1 | `armscat'==3,`c',1)
   display "MNAR param = ( " `c0' " ; " `c1' " )"
   *Modify QoL values
      use "10TT_MI.dta" , clear
      foreach var of varlist qol_3 qol_6 qol_12 qol_18 qol_24 {
         replace `var'=`var'*`c0' if miss_`var'==1 & _mi_m>0 & arm==0
         replace `var'=`var'*`c1' if miss_`var'==1 & _mi_m>0 & arm==1
         }
   *Calculate modified QALY and INB
      mi passive: replace qaly=0.125*qol_0 + 0.25*qol_3 + 0.375*qol_6 + (0.25*1.965)*qol_12 +
(0.5*0.965)*qol_18 + (0.25*0.965)*qol_24
      mi passive: generate inb=qaly*20000-totalcost
   *Do MI analysis and save results
         mi estimate: regress inb arm
         matrix res=r(table)
         post inbgraph (`c') (`c0') (`c1') (`armscat') (res[1,1]) (res[5,1]) (res[6,1])
   }
   }
postclose inbgraph

** Graph set up
   use "inbgraph.dta", clear
   *Reshape to have one row by parameter (and 3 series, by 'armscat')
      drop c0 c1
      tab armscat
      reshape wide inb ll ul, i(c) j(armscat)
      list, noobs
   *Convert to "-%" for presentation
      gen cperc=string(-(1-c), "%8.2f")+"%"
```

```
** Graph
   graph twoway (connected  inb1 c) (connected inb2 c) (connected inb3 c) ///
              (rarea ll1 ul1 c, color(navy%10)) ///
              (rarea ll2 ul2 c, color(maroon%10)) ///
              (rarea ll3 ul3 c, color(forest_green%10)), ///
                yline(0.5,lc(gs5) lw(thin) lpattern(dash)) ///
                text(-4000 1 "Control more cost-effective", placement(w)) text(4000 1 "10TT
more cost-effective", placement(w)) ///
                xtitle("MNAR reduction for imputed QoL")  xlabel(0.80 "-20%" 0.85 "-15%" 0.90
"-10%" 0.95 "-5%"  1.0 "MAR") ///
                ytitle("Incremental Net Monetary Benefit (£)") ylabel(-5000(1000)5000, angle(0)
format(%9.0fc)) ///
                legend(order(1 4 2 5 3 6) label(1 "Reduction applied to both arms") label(2
"Applied to control arm only") label(3 "Applied to intervention arm only") label(4 "95% CI")
label(5 "95% CI") label(6 "95% CI"))
```

2) Contour plot for the probability of 10TT being cost-effective by MNAR parameter

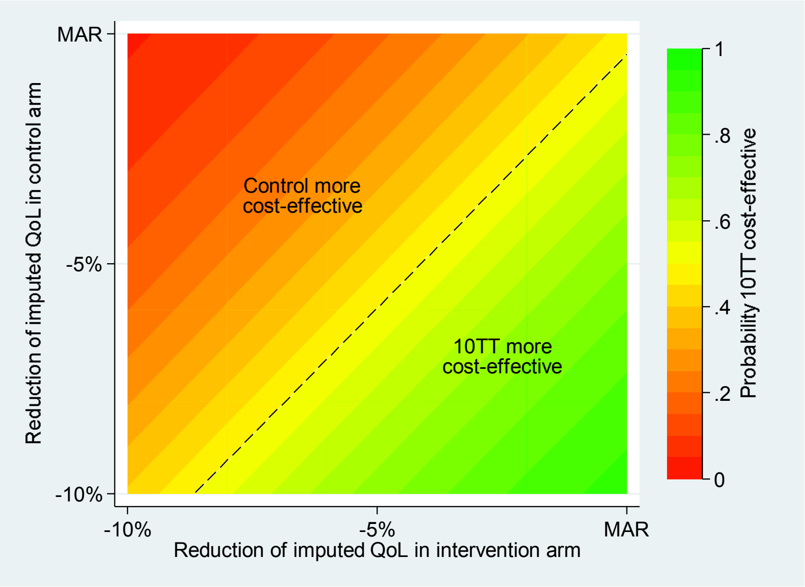

Note: example shown here with probability of being cost-effective, but could also display other results such as INMB or ICER.

<u>Stata code:</u>

```
** Calculate results for different MNAR parameters

postfile contour c0 c1 inmb ll ul pce using "contour_graph.dta", replace  //Set up 'postfile'

forvalues c0=0.9 0.92 to 1.0 {
   forvalues c1=0.9 0.92 to 1.0 {
      *Modify QoL values
         use "10TT_MI.dta" , clear
         foreach var of varlist qol_3 qol_6 qol_12 qol_18 qol_24 {
            replace `var'=`var'*`c0' if miss_`var'==1 & _mi_m>0 & arm==0
            replace `var'=`var'*`c1' if miss_`var'==1 & _mi_m>0 & arm==1
            }
      *Calculate modified QALY and INB
         mi passive: replace qaly=0.125*qol_0 + 0.25*qol_3 + 0.375*qol_6 + (0.25*1.965)*qol_12
+ (0.5*0.965)*qol_18 + (0.25*0.965)*qol_24
         mi passive: generate inb=qaly*20000-totalcost
      *Do MI analysis and save results
         mi estimate: regress inb arm
         matrix res=r(table)
         local pce = normal(res[1,1]/res[2,1])
         post contour (`c0') (`c1') (res[1,1]) (res[5,1]) (res[6,1]) (`pce')
         //Saving INMB, 95%CI, and probability cost-effective (at £20,000/QALY)
      }
```

```
   }
postclose contour

** Graph
** Set up
   use "contour_graph.dta",clear
   label var c0 "MNAR parameter in control arm "
   label var c1 "MNAR parameter in intervention arm"
   label var pce "Probability 10TT cost-effective"

** Graph
   graph twoway (contour pce c0 c1, ccuts(0(0.05)1) zlabel(#6) scolor(red) ecolor(green)) ///

             (contourline pce c0 c1, ccuts(0.5) colorlines lpattern(dash)), ///
             ytitle("Reduction of imputed QoL in control arm") ylabel(0.90 "-10%" 0.95 "-5%" 
1.0 "MAR", angle(0)) ///
             xtitle("Reduction of imputed QoL in intervention arm") xlabel(0.90 "-10%" 0.95 "-
5%" 1.0 "MAR")  ///
             text(0.965 0.935 "Control more" "cost-effective") text(0.93 0.975 "10TT more" 
"cost-effective")
```
